# Supplementary material for: Subtype-Selective Peptide and Protein Neurotoxic Inhibitors of Nicotinic Acetylcholine Receptors Enhance Proliferation of Patient-Derived Glioblastoma Cell Lines
Source: Toxins (Basel). 2024 Feb 2;16(2):80. doi: 10.3390/toxins16020080 (PMC10891657; doi:10.3390/toxins16020080)
Supplement: Supplementary file 1 [file toxins-16-00080-s001.zip › Supplementary_table_S1.pdf]

Table 1. RT-PCR oligonucleotide primers used for detection of *Homo sapiens* nAChR genes expression.

Gene code	Sequence
CHRNA1	forward<br>5' – GGCTCCGAACATGAGACCCG – 3'
	reverse<br>5' – CCACTCCTCAGACGCATTG – 3'
CHRNA3	forward<br>5' – GTGGATGAAGTAAACCAGATCATGGAG – 3'
	reverse<br>5' – GGAACCGAACTTCATGGTACAGTTTTG – 3'
CHRNA4	forward<br>5' – CCTCGGCCTGTCCATCGCTCA – 3'
	reverse<br>5' – AAGACGGTGAGCGACAGCAGC – 3'
CHRNA5	forward<br>5' – ACTGTCACCTGGACTCCACCG – 3'
	reverse<br>5' – AACAGCTGTCGGTTCTGTTTCCTTTG – 3'
CHRNA6	forward<br>5' – TGTGGGCTGTGCAACTGAGGAG – 3'
	reverse<br>5' – CAATGTCGGGCTTCCAAATCTTATC– 3'
CHRNA7	forward<br>5' – CCCGGCAAGAGGAGTGAAAGGT – 3'
	reverse<br>5' – ACCGAGAGGCCCACGATGATC – 3'
CHRNA9	forward<br>5' – AGAGCCTGTGAACACCAATGTGG – 3'
	reverse<br>5' – CTCAGAGCAGCAGCCATAGGAG – 3'
CHRNA10	forward<br>5' – GATGTAGCAGCCTTCCCGTTCG – 3'
	reverse<br>5' – AGCAGCAGCGTGAAGGTGACG – 3'
CHRNB1	forward<br>5' – GTGTCAGGGTCAGGGTTGGT – 3'
	reverse<br>5' – CGACGCTAATGTCCAGAGCC – 3'
CHRNB2	forward<br>5' – TGTACGAGGTGTCCTTCTATTCCAATG– 3'
	reverse<br>5' – TGTAGAAGAGCGGCTTGCGGC – 3'
CHRNB3	forward<br>5' – CGCCGAAAATGAAGATGCCCTCC – 3'
	reverse

	5’ – GTCAGCATTTTCAAAGAACTATGTCAGG– 3’
CHRNB4	forward<br>5’ – CAACAACCTGATCCGCCCAGC – 3’
	reverse<br>5’ – GTTGTAAAGCACGATGTCAGGCAAC – 3’
18S	forward<br>5’ - GGCCCTGTAATTGGAATGAGTC - 3’
	reverse<br>5’ - CCAAGATCCAACTACGAGCTT - 3’
