# Supplementary figures and images for: Subtype-Selective Peptide and Protein Neurotoxic Inhibitors of Nicotinic Acetylcholine Receptors Enhance Proliferation of Patient-Derived Glioblastoma Cell Lines
Source: Toxins (Basel). 2024 Feb 2;16(2):80. doi: 10.3390/toxins16020080 (PMC10891657; doi:10.3390/toxins16020080)

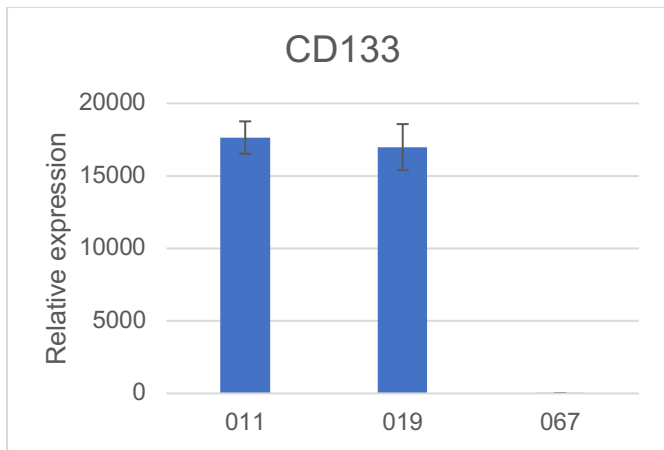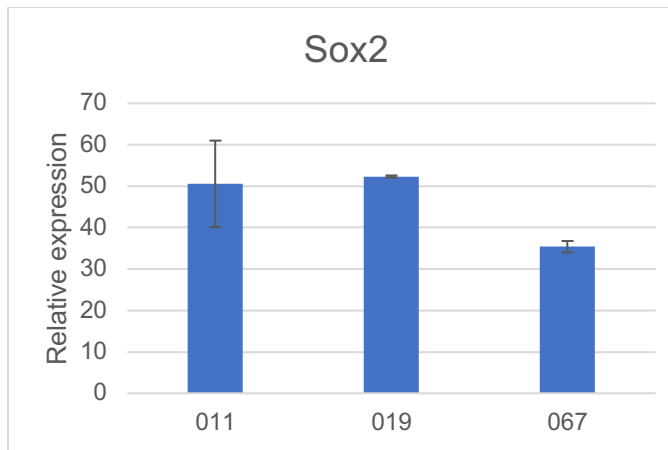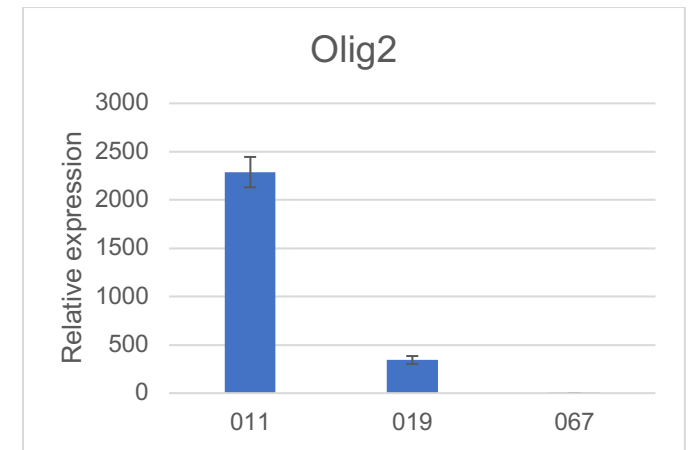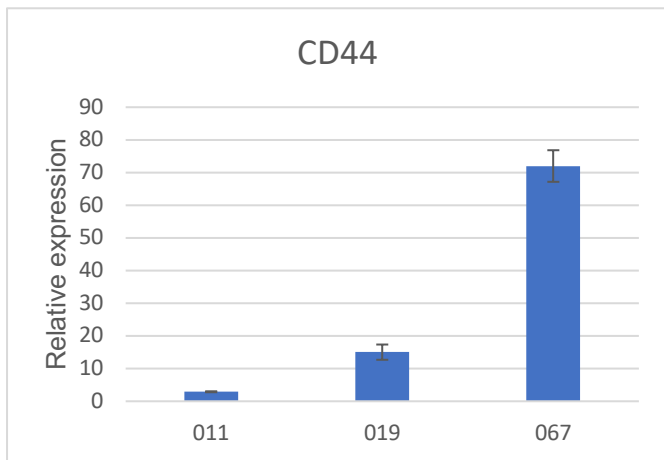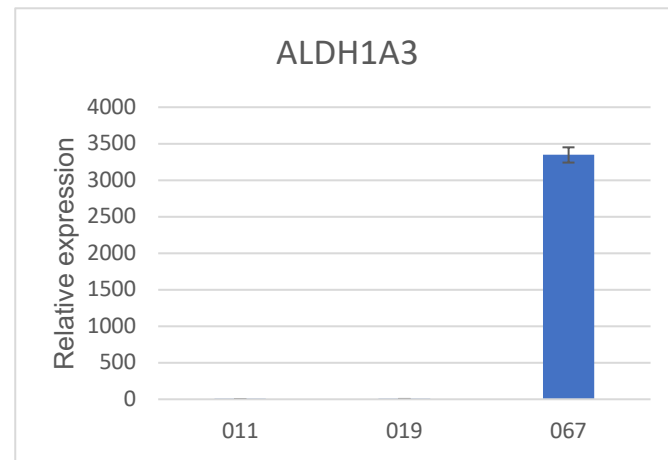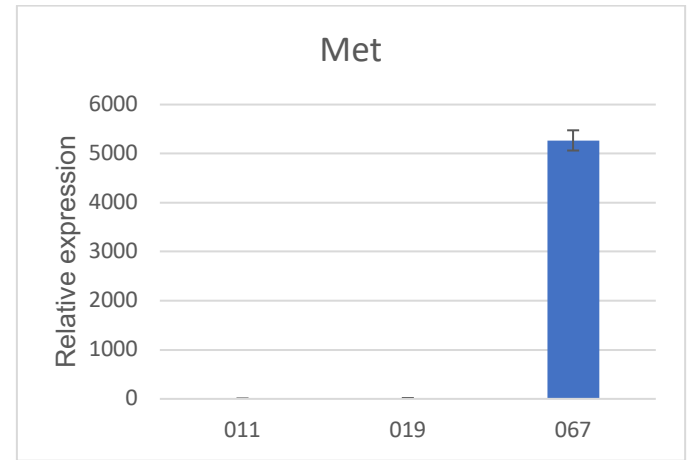

Supplement: Supplementary file 1 [file toxins-16-00080-s001.zip › Figure S3.pdf]

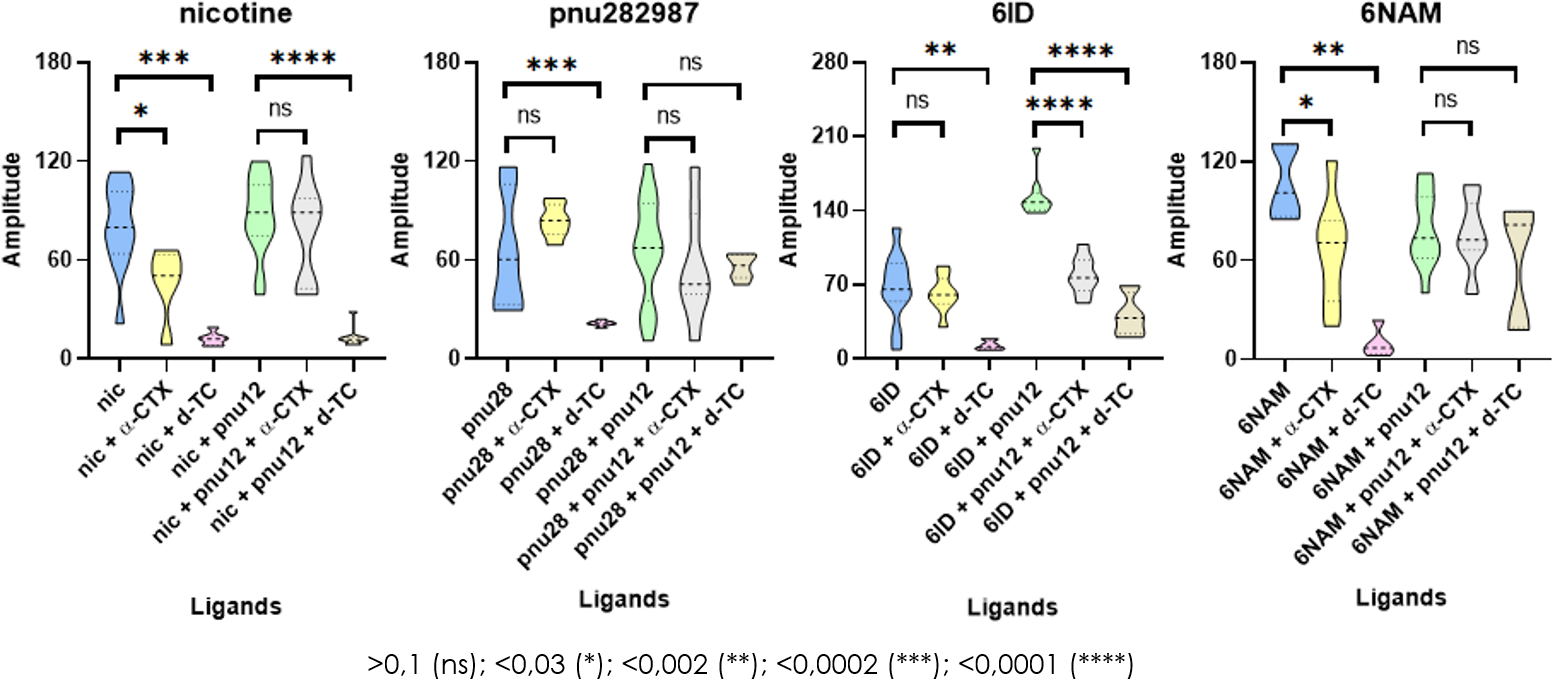

Supplement: Supplementary file 1 [file toxins-16-00080-s001.zip › figure_S1.png]

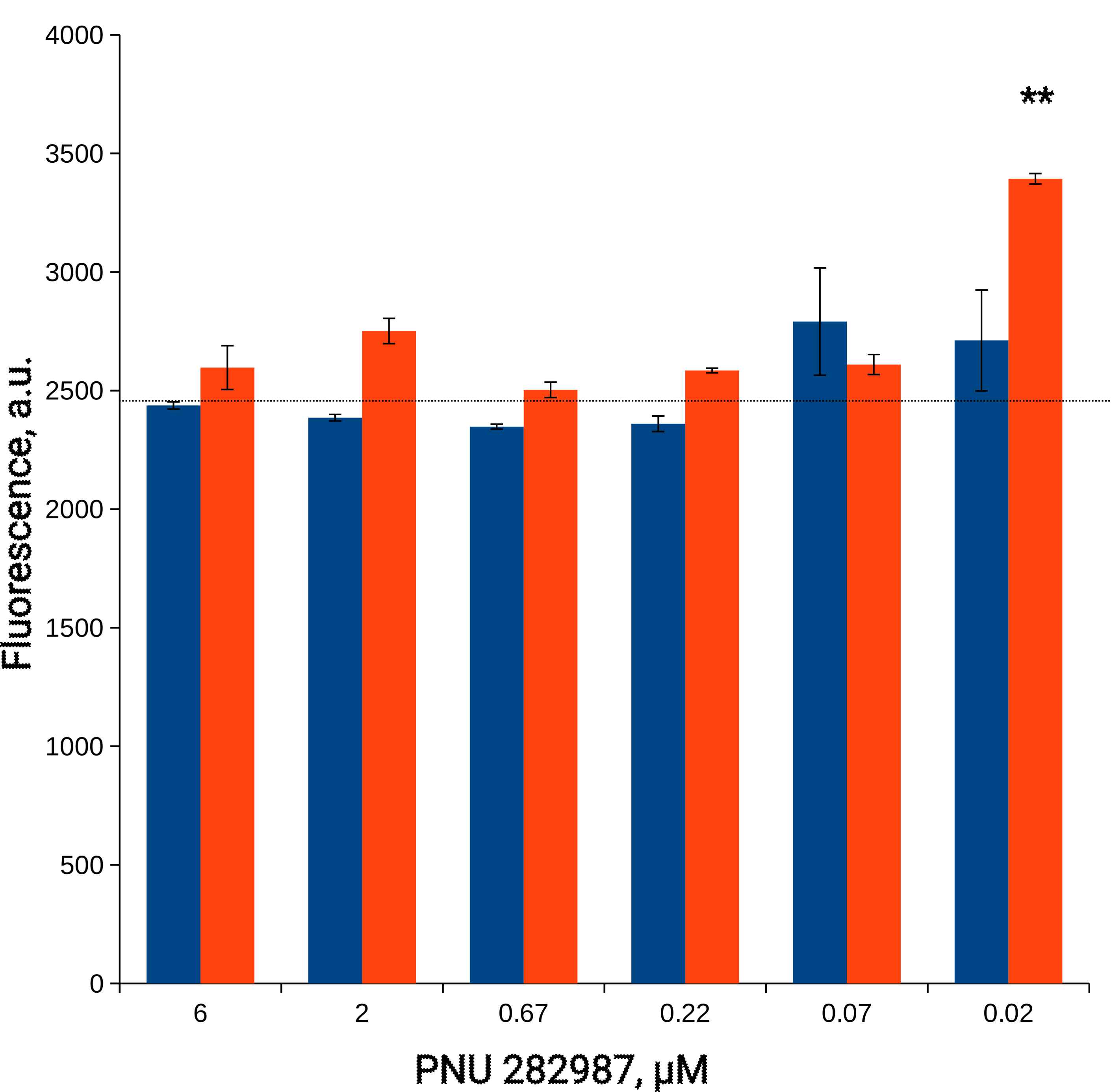

Supplement: Supplementary file 1 [file toxins-16-00080-s001.zip › figure_S2.png]
